# A descriptive analysis of the growth of unrecorded interactions amongst cattle-raising premises in Scotland and their implications for disease spread

**DOI:** 10.1186/s12917-016-0652-5

**Published:** 2016-02-24

**Authors:** Jessica Enright, Rowland R Kao

**Affiliations:** Computing Science and Mathematics, University of Stirling, Stirling, FK9 4LA UK; Institute of Biodiversity, Animal Health, and Comparative Medicine, University of Glasgow, Jarrett Building, Glasgow, G61 1QH UK

**Keywords:** Cattle movement network, Modeling, Scotland

## Abstract

**Background:**

Individual animal-level reporting of cattle movements between agricultural holdings is in place in Scotland, and the resulting detailed movement data are used to inform epidemiological models and intervention. However, recent years have seen a rapid increase in the use of registered links that allow Scottish farmers to move cattle between linked holdings without reporting.

**Results:**

By analyzing these registered trade links as a number of different networks, we find that the geographical reach of these registered links has increased over time, with many holdings linked indirectly to a large number of holdings, some potentially geographically distant. This increase was not linked to decreases in recorded movements at the holding level. When combining registered links with reported movements, we find that registered links increase the size of a possible outward chain of infection from a Scottish holding. The impact on the maximum size is considerably greater than the impact on the mean.

**Conclusions:**

We outline the magnitude and geographic extent of that increase, and show that this growth both has the potential to substantially increase the size of epidemics driven by livestock movements, and undermines the extensive, invaluable recording within the cattle tracing system in Scotland and, by extension, the rest of Great Britain.

## Background

Explicit tracing of livestock movements is becoming increasingly common in many countries. This high level of detail offers opportunities to improve the efficiency and effectiveness of infectious disease surveillance and control, with the potential for substantial cost savings and, by reduction of disease burden, improvement in public and livestock health [1, 2]. Analysis of these detailed records of animal movements as a network is also becoming common, with the terminology and methods of network analysis making an impact on veterinary epidemiology [3, 4].

Since January 2001, it has been legally required to record movements of cattle between holdings in Great Britain. These movements are recorded by the British Cattle Movement Service (BCMS) to allow individual-level tracing of animals for public safety and disease control. This careful recording is consistent with European council directives [5], and is implemented in British and Scottish legislation. These recorded movements are also used as the basis of several epidemiological analyses (examples include [6–9]). When livestock movements are interpreted as a network, it is common to cast the holdings as nodes of the network and movements as arcs between those nodes. For full details of network terminology as used in veterinary epidemiology, we direct the reader to [3, 4].

Because reporting animal movements that are frequent and repetitive can impose a significant administrative burden on farmers, several programs exist to allow regular or short-distance movements, particularly within a single business, to go unreported. One such program is Cattle Tracing System (CTS) Links, which are granted to account for movements between holdings either for the use of shared facilities or for additional land (commonly used for grazing). When a Link is registered, one holding is listed as a main holding, and the other as a secondary holding. Once a link is established, cattle may be moved from the main holding to the secondary holding and back without reporting the movement to BCMS. As with most livestock movements in Scotland, movement of an animal from a CTS Linked holding should trigger a “13-day standstill” on the destination holding – a period of 13 days during which animals (except for those in a specially exempted category) may not be moved away from that destination holding.

Previous work [10] has shown that CTS Links present in 2008 could pose a significant epidemiological risk in a foot-and-mouth disease outbreak, with particular potential to increase the geographic extent of an outbreak. The increased danger posed by CTS Links comes from both the possibility that animals are moved when they would not be if their movement had to be reported, and from the impediment to rapid animal tracing in the case of an outbreak. In addition, because the frequency of link use is not recorded, movements between linked holdings represent an unquantifiable risk, and so could undermine efforts to optimize risk-based surveillance and risk management.

Since 2008 the number and connectivity of CTS Links in Scotland has increased dramatically. Motivated by the potential for CTS Links to contribute to epidemiological risk and the increase in their number since the last significant study of them, we investigate the state of CTS Links in Scotland. Our objective is to characterize the current network of links in Scotland, and explore its growth over time by plotting the change in the geographic distribution of links, and in epidemiologically-relevant network measures.

## Methods

### Data sources and adaptations

Several agricultural datasets were sourced to analyse the current state of the CTS Links network in Scotland. We used the 2010 Agricultural Census to find geographical locations of holdings, and a 2014 extract of individual-level cattle movement records (CTS) from the British Cattle Movement Service to estimate the number of animals on each holding, and for the recorded movements themselves. Information on CTS Links consisting of the pairs of registration numbers of holdings in open Links in December of 2009, 2010, 2012, 2013 and 2014 was provided by Scottish Government.

Some holdings that are in a CTS Link are not listed in the Agricultural Census because not every small holding is recorded in every year’s Agricultural Census. Where possible, we have estimated geographic locations using the county and parish of holdings, by taking a mean easting and northing across holdings in the same county and parish, and perturbing it at random by up to 3 miles. These estimated locations make up less than 5 % of holdings mapped.

### Availability of data and materials

Due to the commercially sensitive nature of the data used in this work, we cannot make the data publically available. The data on CTS Links and the Agricultural Census are held by Scottish Government, and the data on cattle movement records by the British Cattle Movement Service.

### Network analysis

Because we use a number of standard network analysis methods, we briefly review some terms. We generate two types of networks: *directed networks* when using known animal movements with a known direction, and *undirected networks* when using only CTS Links, which have movement in both directions over the Link.

In an undirected network, the *degree* of a node (here a holding) is the number of network neighbours it has (here the number of holdings it is Linked to) A *component* of an undirected network is a set of nodes of the network that are joined up both directly and indirectly. More precisely, two nodes in a network are in the same component if there is some path between them in the network. This concept is epidemiologically useful because in a disease spreading on a network the size of the largest component is an upper bound on the number of holdings infected over that network.

When analyzing a directed network, which includes explicit movement direction we calculate, as described in [3], the size of an *infection chain* from a holding, which is the total number of other holdings in the network that could be infected by that holding, either directly or indirectly. We also use a similar notion: the size of an *infector chain* of a holding, which is the total number of other holdings in the network that could infect that holding, either directly or indirectly. The infector chain is, in some sense, a backwards version of the infection chain. These two measures are an indicator of the susceptibility of the network to disease [3].

All analyses were performed using Python code written by the authors, making use of **networkx** and **matplotlib** libraries.

## Results and discussion

### Networks investigated

Our analysis includes 41 different networks: five undirected networks, each composed only of the Links in one of our five study years, and 36 directed networks, three for each month of 2014: one composed of that month’s reported cattle movements in Scotland, a second composed of that month’s reported movements with simulated movements along the 2014 CTS Links added in, and a third derived from the network of reported movements by contracting all holdings directly or indirectly joined-up by Links in 2014 into a single super-holding. This extreme contraction is equivalent to assuming that there is constant movement along all links, and is the approach predominantly used by Orton et al. [10].

When adding in links to reported movements, we add a movement from the main holding to the secondary holding direction on each link on the 14th of the month, and one returning on the 21st. There are no data currently available on the regularity or timing of use of CTS Links, so this timing of inclusion should be considered only as a demonstration.

### Characterizing the network in combination with recorded movement

When we consider the combination of all the CTS Links into a network, we see that not only are holdings linked to each other directly by their individual links, but that the links chain together to form larger components: thus a holding with only a small number of CTS Links may be indirectly linked to a large number of other holdings in a component. Because unrecorded animal movement and therefore pathogen movement is possible throughout a component, the size and geographic extent of these components is one of our primary interests.

**Fig. 1:**
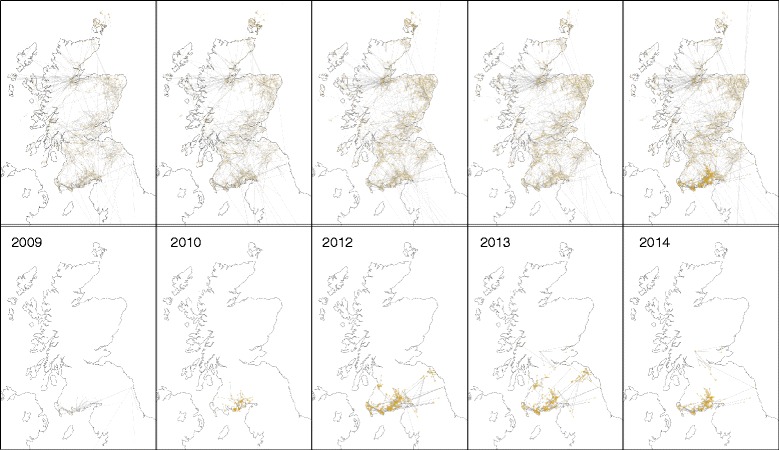
In the top row maps of the locations of linked holdings in Scotland in December 2009, 2010, 2012, 2013, and 2014, and in the bottom row maps of the holdings in the largest connected components of links in December 2009, 2010, 2012, 2013, and 2014

We have used the Agricultural Census and each of five years of CTS Links information to plot the maps of links shown in Fig. 1. As seen in the top row of Fig. 1, the overall network has grown substantially in size and connectivity since 2009. Both long-range links that connect geographically distant holdings and short-range links that densely cover areas with high concentrations of cattle holdings have increased. Throughout the time period of study short-range links are more common than long-range links: in 2014, approximately 65 % of links were between holdings within five miles of each other.

In the bottom row of Fig. 1, we see that the geographic extent of the largest component has increased dramatically over time, with a large increase between 2010 and 2012. The number of holdings within 3 and 10 km of a holding in the largest component in each of our study years is shown in Table 1. The size of the network increased consistently from 2009 to 2013, but, surprisingly, decreased slightly from 2013 to 2014. The size of the largest component has shown an overall increasing trend, but has decreased slightly between 2012 and 2013, and again between 2013 and 2014.

**Table 1:** The number of cattle holdings within 3 and 10 km of a holding that is in the largest CTS Links component in each of 2009, 2010, 2012, 2013, and 2014

	Number of holdings within distance of a holding in the largest component	
Year	3 km	10 km
2009	1,265	3,437
2010	1,413	3,532
2012	3,380	6,691
2013	3,697	10,153
2014	2,356	5,561

**Fig. 2:**
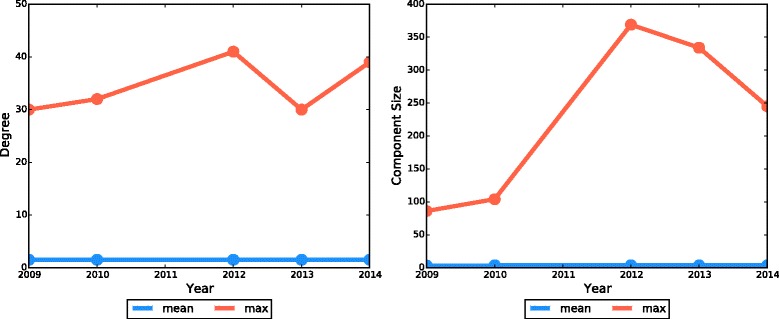
Mean and maximum degree (left) and component (right) size change for Scottish holdings in the CTS Links networks over Decembers of 2009, 2010, 2012, 2013, and 2014

In Fig. 2 we plot degrees and component sizes for the CTS Links network in each of our five study years, reporting the mean and maximum for each.

While the largest component has grown over time to 334 in 2013, and 245 in 2014 from less than 100 in 2009, the mean size of a component has not increased over the years.

Neither the mean nor the maximum number of holdings a holding is linked to (its *degree*) has changed substantially over our study period. Thus when looking at any single holding in the CTS Links network, the situation in 2009 looks much the same as in 2013. It is only when we consider the overall picture that we see a change.

We investigated changes in reported movement volume for holdings without CTS Links, and found no relationship between the establishment of a link and any change in reported movements. It does not appear to be the case that a holding that establishes a CTS Link then reports fewer movements the following year.

We have calculated the size of infection and infector chains for holdings within each month of 2014,for the directed networks described above: the network of only reported movements without CTS Links, the network of reported movements with movements along CTS Links added in, and the network of reported movements with holdings in the same links component contracted to a superholding.

**Fig. 3:**
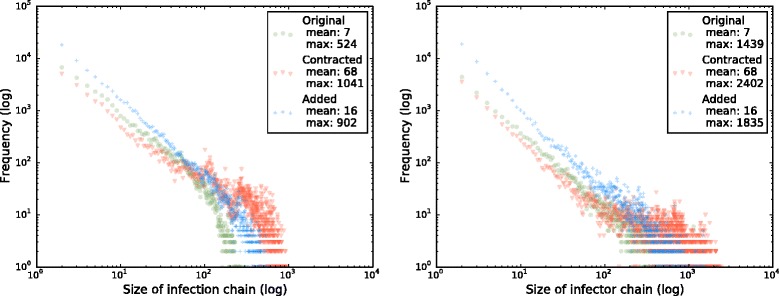
Frequencies of infection (left) and infector (right) chain sizes for networks of Scottish cattle movements within each month of 2014 without CTS Links (green circles), with movements on CTS Links added in (blue crosses), and with holdings directly or indirectly connected by CTS Links contracted to a single holding (red triangles)

In Fig. 3 we see that sizes of infection and infector chains increase when CTS Links are added to recorded movements, with an even larger change in the maximum of these numbers than in the mean. The change in these numbers highlights the potential danger in CTS Links, both for disease spread and for legal traceability. Without CTS Links cattle holdings in Scotland could have received infection from or transmitted it to a mean of 7 holdings within the average month. With CTS Links added, this more than doubles to 16 holdings. When all holdings linked by a chain are aggregated into a super-holding, this number increases more dramatically to 68.

This increase impacts not only holdings that are members of a CTS Link, but also those that are not via animal movement chains that involve holdings that are in CTS Links. The mean size of an infection chain over all holdings represents an estimate of the number of holdings that would have to be investigated using tracing in an outbreak: this number would increase significantly if fenceline contacts were taken into account.

### Focus on the largest component

As an example of the growth of the system, we focus our attention on the largest connected component in 2014, and examine its development over several years.

**Fig. 4:**
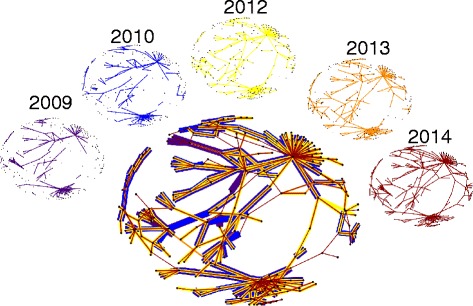
Topological growth of the largest chain in 2014. Dots representing the 245 holdings in the largest chain in 2014 are shown in the same layout for each year. We show the CTS Links present in each of 2009, 2010, 2012, 2013, and 2014 across the top, with all links from those years shown together in the large network in the centre. The colours of links are consistent between the small networks and the large network, where the links are overlaid with more recent years on top of older links. Note that while most links that appear in a year occur also in subsequent years, some do not, and will therefore have cool coloured links not overlaid with warmer coloured links in the central aggregate network

As we can see in Fig. 4, the largest component in 2014 formed over a number of years, not just by addition of single holdings joining a larger component, but also by several smaller connected components being joined together by the addition of new CTS Links. It is noticeable that most links seem to persist over several years, though a few occur do not, and therefore appear only in cooler colours in the central aggregate network of Fig. 4. Most of these links are issued only for a single year, and so persistent links are renewed by the farmer every year. We also see that, while there are a small number of holdings in the largest connected component with many links, the majority of the holdings have only a small number of links, and removing the holdings with a large number of links would decrease the size of the component, but would still leave the majority of holdings in that component joined-up.

## Conclusions

The development of detailed records of livestock movements provides important opportunities to implement risk-based surveillance and testing, but is dependent on available data being sufficiently unbiased to make analyses of these data robustly predictive. Here we show that exemptions from recording in GB have the potential to compromise this robustness, and that their usage has been growing year-on-year from 2009 to 2013.

Despite the fact that the average number of holdings a linked holding is linked to has not changed over time, the large increase in the number of holdings involved in links has lead to an alarming growth in the overall CTS Links network in Scotland. The largest connected component in the current network reflects this effect: it was formed by a small number of links joining up relatively small components, and could be disassembled into smaller, more manageable components by the removal of only a few links. However, these link removals would have to be highly strategic: simply removing links to holdings with a large number of links would not be adequate.

An important consequence of the dramatic increase in link usage is the possibility of increased disease spread or more widespread tracing required in an outbreak is substantial: the mean number of holdings in a possible outward infection chain from a single holding within a month more than doubles when CTS Links are taken into account, increasing even for holdings that are not directly involved in a CTS Link. This network growth undermines the extensive, invaluable recording within the cattle tracing system in Scotland and, by extension, the rest of Great Britain. However, our investigations show that its impact could be mitigated by the removal of relatively few links.

Monitoring links on a holding-by-holding level would not have revealed the growth of this network, and so for disease control and robust traceability, this network should be monitored as a system. The overall picture is one of a system that has become more than the sum of its parts: while most links are in themselves not a large epidemiological risk, when combined into an overall network the potential for disease spread or traceability failure is significant.

## Abbreviations

BCMS: British Cattle Movement Service
CTS: Cattle tracing system
GB: Great Britain
